# Generation of a chemical genetic model for JAK3

**DOI:** 10.1038/s41598-021-89356-4

**Published:** 2021-05-12

**Authors:** Judit Remenyi, Rangeetha Jayaprakash Naik, Jinhua Wang, Momchil Razsolkov, Alyssa Verano, Quan Cai, Li Tan, Rachel Toth, Samantha Raggett, Carla Baillie, Ryan Traynor, C. James Hastie, Nathanael S. Gray, J. Simon C. Arthur

**Affiliations:** 1 Division of Cell Signalling and Immunology, School of Life Sciences, Wellcome Trust Building, University of Dundee, Dundee, DD1 5EH UK; 2 Department of Cancer Biology, Dana Farber Cancer Institute, Department of Biological Chemistry and Molecular Pharmacology, Harvard Medical School, Boston, USA; 3 MRC PPU Reagents and Services, School of Life Sciences, University of Dundee, Dundee, DD1 5EH UK

**Keywords:** Cytokines, Immunology, Cytokines, Kinases, Medicinal chemistry

## Abstract

Janus Kinases (JAKs) have emerged as an important drug target for the treatment of a number of immune disorders due to the central role that they play in cytokine signalling. 4 isoforms of JAKs exist in mammalian cells and the ideal isoform profile of a JAK inhibitor has been the subject of much debate. JAK3 has been proposed as an ideal target due to its expression being largely restricted to the immune system and its requirement for signalling by cytokine receptors using the common γ-chain. Unlike other JAKs, JAK3 possesses a cysteine in its ATP binding pocket and this has allowed the design of isoform selective covalent JAK3 inhibitors targeting this residue. We report here that mutating this cysteine to serine does not prevent JAK3 catalytic activity but does greatly increase the IC50 for covalent JAK3 inhibitors. Mice with a Cys905Ser knockin mutation in the endogenous JAK3 gene are viable and show no apparent welfare issues. Cells from these mice show normal STAT phosphorylation in response to JAK3 dependent cytokines but are resistant to the effects of covalent JAK3 inhibitors. These mice therefore provide a chemical-genetic model to study JAK3 function.

## Introduction

JAK-STAT (Signal transducer and activator of transcription) signalling pathways are critical to the function of cytokines that act via type I and II cytokine receptors and, as a result, play major roles in controlling the development, homeostasis and activation of immune cells^1,2^. This has led to considerable interest in targeting JAKs to treat immune mediated disease with 5 JAK inhibitors gaining FDA approval for human clinical use with more compounds in development^3–7^. JAKs are a family of 4 non receptor tyrosine kinases consisting of JAK1, JAK2, JAK3 and TYK2 (Tyrosine Kinase 2) which act predominantly via the phosphorylation of STAT transcription factors. Seven mammalian genes for STATs exist and their phosphorylation by JAKs leads to their dimerization and translocation to the nucleus where they drive the activation of various STAT dependent genes. JAK-STAT pathways mediate the effects of multiple cytokines with diverse effects on the immune system. This diversity is facilitated by the ability of different receptors to recruit specific JAK and STAT isoforms as well as differences in the feedback control mechanisms activated downstream of receptor activation^1,2^. The importance of JAK-STAT signalling to immunity has been underscored by the finding of a number of clinical mutations in the pathway that lead to immune defects. For example, mutations in JAK dependent cytokine receptors and individual STATs have been linked to increased risk of infection (reviewed in^8^). Loss of function mutations in JAK3 result in Severe Combined Immunodeficiency Disease (SCID), as do mutations in the common gamma chain receptor which recruits JAK3^9–11^ while activating mutations in JAK2 are a frequent cause of myeloproliferative disorders such as myelofibrosis and polycythaemia^12,13^.

Both JAK1 and JAK2 are expressed in immune and non-immune cells and their deletion in mice results in perinatal and embryonic lethality respectively^14–16^. In contrast, JAK3 knockout mice are viable although they show defects in T and B cell development similar to SCID^17–19^. This led to the proposal that selectively targeting JAK3 may be beneficial for the treatment of autoimmune disorders^7^. Together with the role of JAK2 in myeloproliferative disorders this led to the development of multiple JAK inhibitors as potential therapeutics. Ruxolitinib was the 1^st^ to obtain FDA approval in 2011 for myelofibrosis^12^. This was closely followed by Tofacitinib, which was approved in 2012 for rheumatoid arthritis that could not be treated with methotrexate^20^. While both are highly selective for JAKs compared to other kinases, both Ruxolitinib and Tofacitinib can inhibit multiple JAK isoforms and this may contribute to some of the side effects associated with their clinical use such as increased risk of infections and alterations in white blood cell numbers^6,7,21^. Their success has however driven the development of a 2nd generation of JAK inhibitors with the aim of improving selectivity amongst different JAKs, 3 of which have now obtained FDA approval for clinical use. Baricitinib and Fedratinib, which show selectivity for JAK2 over JAK3, have gained approval for myelofibrosis while Upadacitinib, which has selectivity for JAK1, has obtained approval for the treatment of rheumatoid arthritis. In addition, multiple other JAK inhibitors have entered in clinical trials^3–7^. Despite the many advances in this area, it is not fully resolved what the ideal selectivity profile of a JAK inhibitor would be in order to maximise efficacy while maintaining an adequate safety profile. In the context of autoimmune disorders this is likely to be influenced by the predominant cytokines driving the disease.

Most of the JAK inhibitors developed to date target the ATP binding pocket in kinase domain of JAKs and act in an ATP competitive manner. The conserved nature of the ATP binding pocket in JAKs makes obtaining selectivity between isoforms a difficult but not impossible task. An unusual feature of JAK3 is that it contains a cysteine residue 7 residues after the gatekeeper site in the ATP binding pocket while the equivalent position in the other JAK isoforms is a serine. In addition to JAK3, only 10 other mammalian kinases have a cysteine at the position^22^, so inhibitors using an electrophilic warhead to covalently modify this site have the potential to be very selective for JAK3. Several studies have reported JAK3 inhibitors that exploit this idea and using this method it has been possible to develop highly selective and potent JAK3 inhibitors^23–32^.

Small molecule kinase inhibitors have provided valuable insights into signal transduction, a caveat to their use is that even the most selective inhibitor may have off target effects in the cell. One approach to mitigate against this is to generate mutant forms of the kinase that retain activity but are insensitive to the inhibitor. An early example of this approach was the mutation of the gatekeeper site in the ATP binding pocket of p38α or β MAPK (Mitogen Activated Protein Kinase) to a methionine that prevented the binding of the p38 inhibitor SB203580^33^. Knockin mice carrying these mutations were subsequently used to demonstrate the efficacy of p38 inhibitors in autoimmune models was due to inhibition of p38α and not p38β^34^. An analogous approach which has also been extensively used is to engineer the ability to use ATP analogues or to allow them to be targeted by specific inhibitors^35,36^.

The inhibition of JAK3 by covalent inhibitors comes from two components. First reversible ATP competitive binding of the inhibitor to the ATP pocket of JAK3 occurs. This orientates the electrophilic warhead with Cys905 allowing covalent modification of the cysteine via a Michael addition reaction. Mutation of JAK3 to change this cysteine to serine would prevent this covalent modification and should result in increase in the IC50 values for the inhibitor against the mutated JAK3. Cells expressing JAK3 with this Cys to Ser mutation should therefore be resistant to covalent JAK3 inhibitors, providing a way of validating that effects of these compounds are on target. To evaluate this approach, we report here the generation of a JAK3 Cys905Ser knockin mouse and show that cells from these mice exhibit reduced inhibition of JAK3 dependent cytokine signalling by covalent JAK3 inhibitors.

## Results

### Mutation of Cys905 to serine reduces inhibition by covalent JAK3 inhibitors

**Figure 1:**
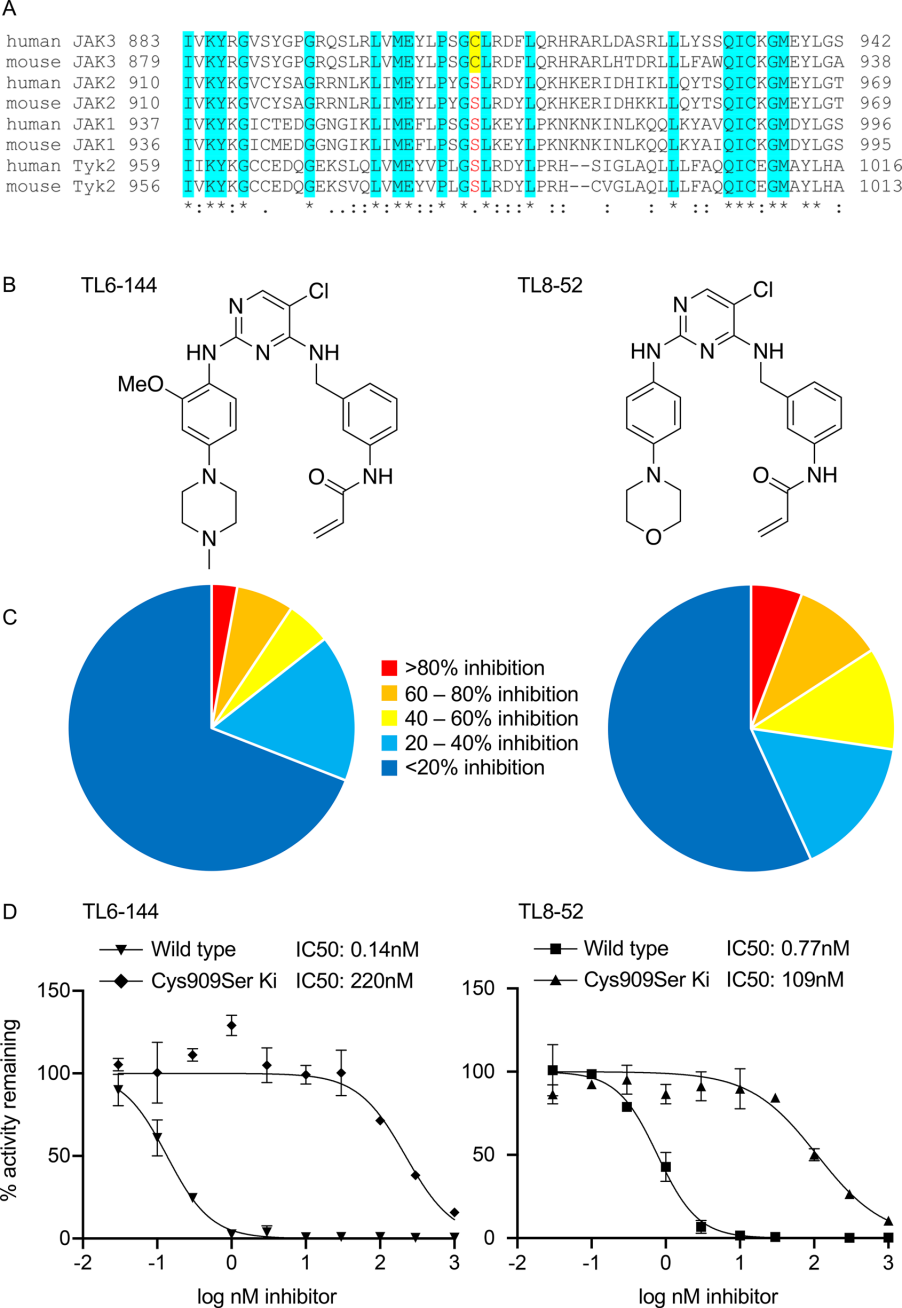
Inhibition of wild type and Cys909Ser mutant JAK3 by TL8-52 and TL6-144. (**A**) Sequence alignment of human and mouse JAK family members around the cysteine residue (Cys909 in human, Cys905 in mouse) in the ATP binding pocket of JAK3. (**B**) Chemical structures of TL6-144 and TL8-52. (**C**) TL6-144 and TL8-52 were screened against a panel of 140 kinases in vitro at 1 μM. The proportion of kinases showing different percentage inhibition is shown in the pie charts. Data for individual kinases is shown in supplementary Table 1. (**D**) TL6-144 and TL8-52 were tested at different concentrations for their ability to inhibit the kinase domain of either wild type JAK3 or a Cys909Ser mutation as described in the methods. Graphs show mean and standard deviation, n = 2.

In JAK3 the amino acid 7 residues after the gatekeeper site in the ATP binding pocket is a cysteine (Cys909 in human JAK3, Cys 905 in murine JAK3), however this position is a serine in all other JAK isoforms (Fig. 1A). This would suggest that a cysteine to serine mutation at this position in JAK3 would be tolerated and not disrupt catalytic activity. To test this, the wild type catalytic domain and a Cys909Ser mutant were expressed and purified from insect cells. The Km for ATP binding was found to be 52.6 μM for the wild type protein and 53.4 μM for the mutant (supplementary Fig. 1). As the mutation of this cysteine residue would be expected to increase the IC50 for covalent JAK3 inhibitors targeting this residue, we tested inhibition of the wild type and mutant kinase domain using two nM covalent JAK3 inhibitors, TL6-144 and TL8-52 (Fig. 1B). Both of these inhibitors are selective for JAK3 with TL6-144 showing > 180 fold selectivity for JAK3 over JAK1 and 2 and TL8-52 showing > 12 fold selectivity^25^. Both compounds also showed specificity for JAK3 over other kinases when assayed at 1 μM against a panel of 139 kinases in vitro, TL6-144 only inhibited 4 other kinases by more than 80% while TL8-52 inhibited 8 (Fig. 1C, supplementary Table 1). Against the wild type JAK3 kinase domain TL6-144 and TL8-52 gave IC50 values of 0.14 and 0.77 nM respectively. These values are at the limit of the resolution of the assay as at this point the inhibitor concentration start to approach the concentration of JAK3 in the assay. When assayed against wild type JAK3 kinase domain or Cys909Ser mutant, both inhibitors showed at least 100-fold increase in the IC50 for the mutant JAK3 compared to the wild type enzyme (Fig. 1D). This would be consistent with the ability of the inhibitors to target JAK3 being potentiated by their ability to covalently modify the cysteine in the ATP binding pocket of JAK3.

### Characterisation of JAK3 Cys905Ser knockin mice

**Figure 2:**
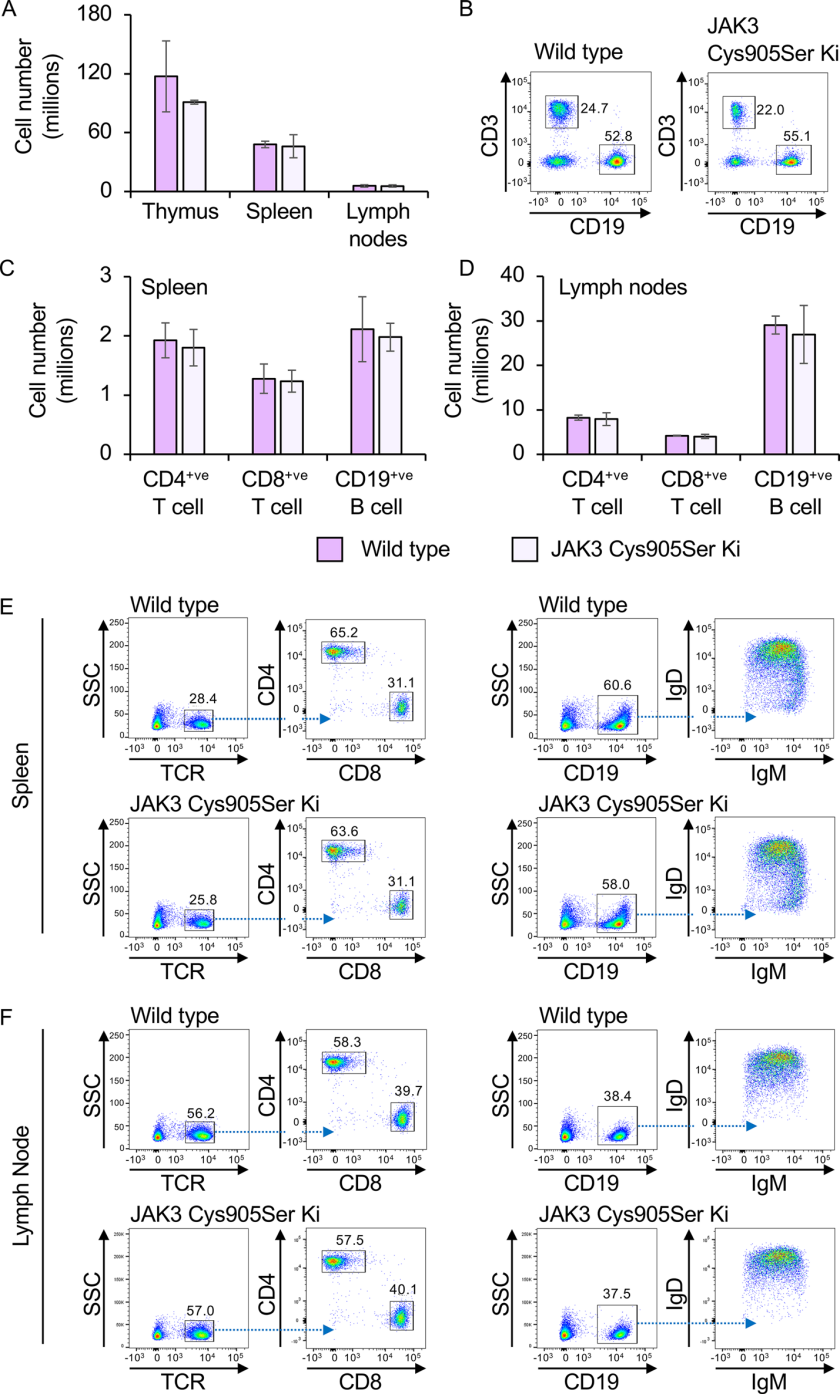
T and B cell numbers in JAK3 Cys905Ser knockin mice. (**A**) Single cells suspensions were generated from the thymus, spleen and lymph nodes from wild type and JAK3 Cys905Ser knockin mice, and red blood cells removed from the splenocytes by RBC lysis as described in the methods. (**B**) Blood was isolated and following RBC lysis stained for CD3 and CD19 to identify T and B cells respectively. Representative plots for wild type and JAK3 Cys905Ser knockin mice are shown. (**C**) Splenocytes were stained for TCRβ, CD4 and CD8 to identify T cells or CD19, IgM and IgD to identify B cells. Numbers of CD4 and CD8 positive T cells and CD19 positive B cells for the spleen are shown. As (**C**) except cells isolated form the lymph nodes were stained. In **A**, **B** and **D** graphs show mean and standard deviation, n = 2 animals per genotype. (**E**) Representative plots showing TCRβ expression with CD4 and CD8 staining in TCRβ positive cells (left panels) or CD19 expression with IgM and IgD staining in CD19 + ve cells (right panels) in spleen. (**F**) As (**E**) except staining is for cells from the lymph nodes.

A Cys905Ser mutation in the endogenous JAK3 gene was generated using CRISPR/Cas9 mediated targeting as described in the methods. Homozygous JAK3 Cys905Ser knockin mice were born at close to the expected Mendelian frequency from heterozygous mattings (31%, n = 89) and the homozygous knockin mice exhibited no overt adverse effects on welfare. The mutation of Cys905 to serine in JAK3 would not be expected to affect JAK3 activity and therefore not be expected to result in the phenotypes seen in JAK3 knockout mice. JAK3 knockout in mice results in reduced thymocyte numbers, a block in B cell development and the pre-B stage and severe B and T cell lymphopenia^17–19^. In contrast, the JAK3 Cys905Ser knockin mice had normal cell numbers in the thymus, spleen and lymph nodes (Fig. 2A). Similar percentages of CD3 + ve T cells and CD19 + ve B cells were present in wild type and JAK3 Cys905Ser knockin blood (Fig. 2B). The knockin mice also showed normal numbers of CD4 and CD8 positive T cells in the spleen and lymph nodes (Fig. 2C–F). B cells were also present at the expected numbers in the spleen and lymph nodes from the JAK3 Cys905Ser knockin mice (Fig. 2C,D). IgM and IgD surface staining was also similar between the wild type and Cys905Ser knockin B cells (Fig. 2E,F). This would suggest that the JAK3 Cys905Ser protein is expressed and is active as the knockin mice do not exhibit the severe lymphopenia seen in JAK3 knockout animals.

### Selective inhibition of JAK3 blocks IL-4 induced signalling in macrophages

**Figure 3:**
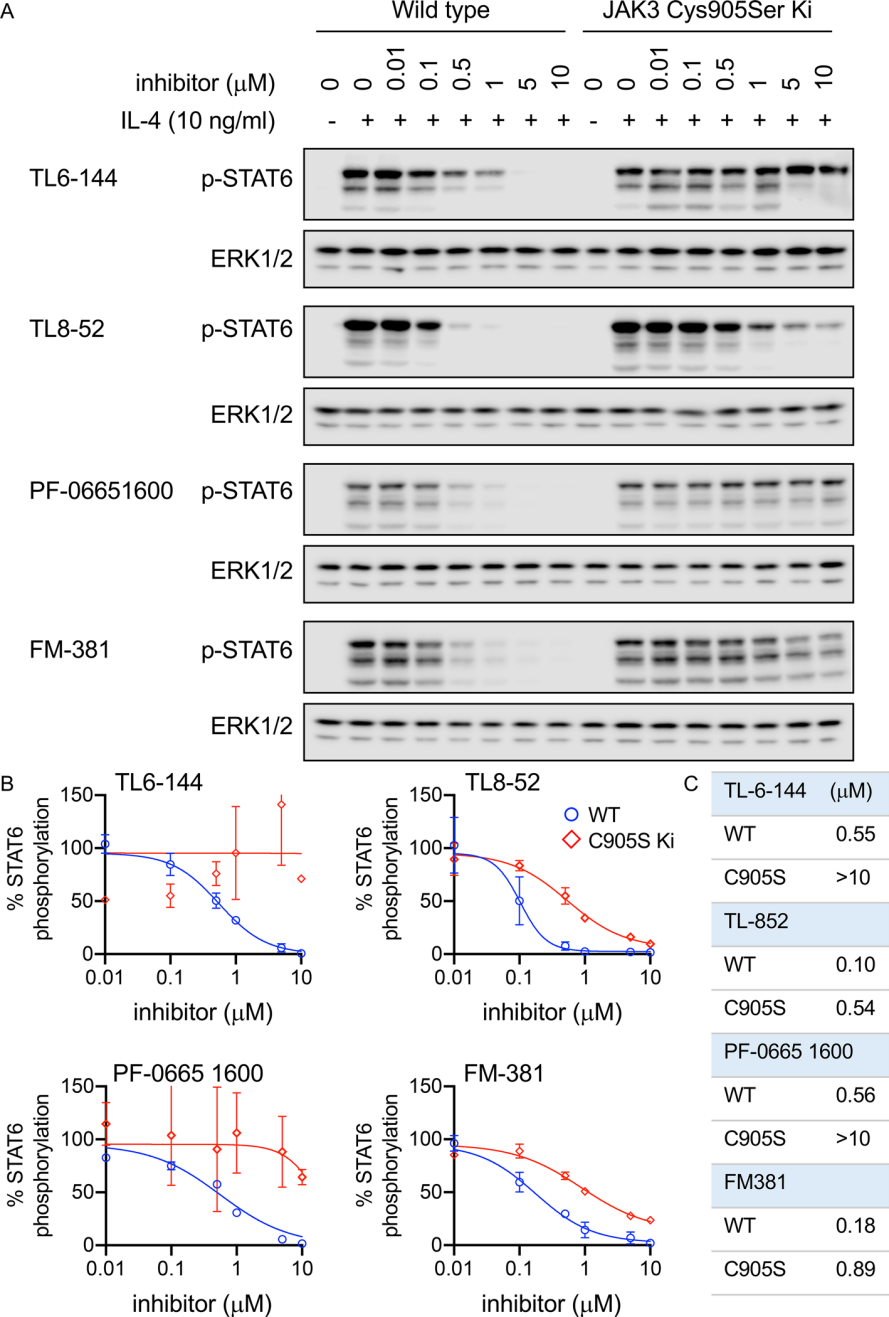
Effect of JAK3 inhibitors on IL-4 induced STAT6 phosphorylation. BMDMs were isolated from wild type and JAK3 Cys905Ser knockin mice. Cells were incubated with the indicated concentrations of inhibitor before stimulation for 30 min with 10 ng/ml IL-4. Cells were lysed and the levels of STAT6 phosphorylated at Tyr641 were assessed by immunoblotting. Levels of ERK1/2 were determined as a loading control. Data is shown for the JAK3 inhibitors TL6-144, TL-852, PF-06651600 and FM-381. Representative blots are shown in (**A**) and quantification of two independent experiments is shown in (**B**) and calculated IC50 values in (**C**).

IL-4 induces the alternative activation of macrophages resulting in cells which promote tissue repair and fibrosis. IL-4 can potentially signal via two receptor complexes, the type I IL-4 receptor consisting of the IL-4 receptor and the common gamma chain and the type II IL-4 receptor complex consisting of the IL-4 receptor and the IL-13 receptor α protein. As the type II receptor, but not the type I receptor also responds to IL-13, the phosphorylation of STAT6 was determined in BMDMs in response to IL-4 and IL-13^37^. This showed that IL-4 induced a much stronger phosphorylation of STAT6 than IL-13, suggesting that IL-4 was acting predominantly via the type I IL-4 receptor in BMDMs (supplementary Fig. 2). In BMDMs, both TL6-144 and TL8-52 were able to block IL4- induced STAT6 phosphorylation in wild type BMDMs with IC50s of 0.55 and 0.1 μM respectively (Fig. 3). In BMDMs from JAK3 Cys905Ser knockin mice the TL6-144 did not inhibit IL-4 induced STAT6 phosphorylation at 10 μM. The IC50 value for TL8-52 was 0.54 μM, approximately 5 times higher than in wild type cells (Fig. 3A,B). In addition to TL6-144 and TL8-52, two recently published covalent JAK3 inhibitors, PF-06651600^24^ and FM-381^23^ were tested (Fig. 3). In both cases, the IC50 for STAT6 phosphorylation was higher in JAK3 Cys905Ser knockin cells compared to wild type cells (> 10 vs 0.56 μM for PF-06651600 and 0.89 vs 0.18 μM for FM381).

**Figure 4:**
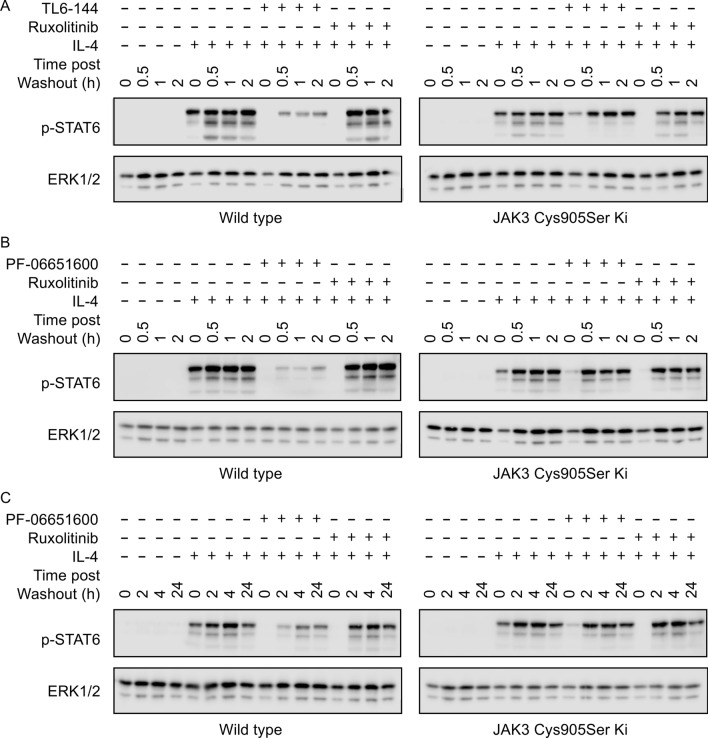
Effect of inhibitor washout on JAK3 inhibition. BMDMs were isolated from wild type and JAK3 Cys905Ser knockin mice. Where indicated cells were incubated with Ruxolitinib (**A**–**C**), TL6-144 (**A**) or PF-06651600 (**B**, **C**) for 2 h. The macrophages were then either stimulated with 10 ng/ml IL-4 for 30 min or washed 3 times in PBS and then incubated in media without inhibitor for the indicated times. The cells were then stimulated with IL-4 for 30 min and STAT6 phosphorylation determined by immunoblotting using levels of ERK1/2 as a loading control. The inhibitor concentrations used were 0.5 μM for Ruxolitinib, 10 μM for TL6-144 and 100 μM for PF-06651600.

Covalent inhibitors would be predicted to have very slow off rates and therefore inhibition should remain even if the unbound inhibitors are washed out of the cells. To test this, cells were treated with a high concentration of inhibitor for 2 h before being washed 3 times in PBS and then incubated in media without inhibitor for 0.5 to 24 h. If the inhibitor acts via a non-covalent mechanism, inhibition would not be expected to remain following washout from the cells. Ruxolitinib is a non-covalent pan-JAK inhibitor that can inhibit multiple JAK-STAT pathways in macrophages^21^. When cells were stimulated with IL-4 before washout, Ruxolitinib was able to completely block STAT6 phosphorylation. Inhibition was however completely lost when the cells were IL-4 stimulated 30 min after Ruxolitinib washout, consistent with the non-covalent mechanism of inhibition (Fig. 4A). In similar experiments, 10 μM TL6-144 completely blocked IL-4 induced STAT6 phosphorylation in the absence of any washout. When cells were stimulated with IL-4 from 30 min to 2 h after inhibitor washout some STAT6 phosphorylation was still seen, however this was much lower than in control cells that had not been pre-treated with TL6-144 (Fig. 4A). While this is consistent with TL6-144 covalently modifying JAK3 it could also be explained by inhibitor being retained in the cell independently of JAK3 binding during the washout steps. To confirm this was not the case, similar experiments were done in JAK3 Cys905Ser knockin cells. In these cells, where TL6-144 can no longer covalently tag JAK3, washout of TL6-144 resulted to the complete loss of inhibition of STAT6 phosphorylation (Fig. 4A). Similar experiments were also carried out with PF-06651600. Similar to TL6-144 inhibition of STAT6 phosphorylation in wild type cells was retained following washout of PF-06651600 (Fig. 4B). As the level of inhibition was lower at 2 h post washout than 30 min the experiment was repeated to look at 4 and 24 h time points for the washout step. Some inhibition was retained at the 24 h time point however this was less than observed at 2 h (Fig. 4C). As for TL6-144, in JAK3 Cys905Ser knockin cells inhibition of STAT6 phosphorylation was not retained at any time post washout (Fig. 4B,C).

### Covalent JAK3 inhibitors do not block JAK3 independent cytokines

**Figure 5:**
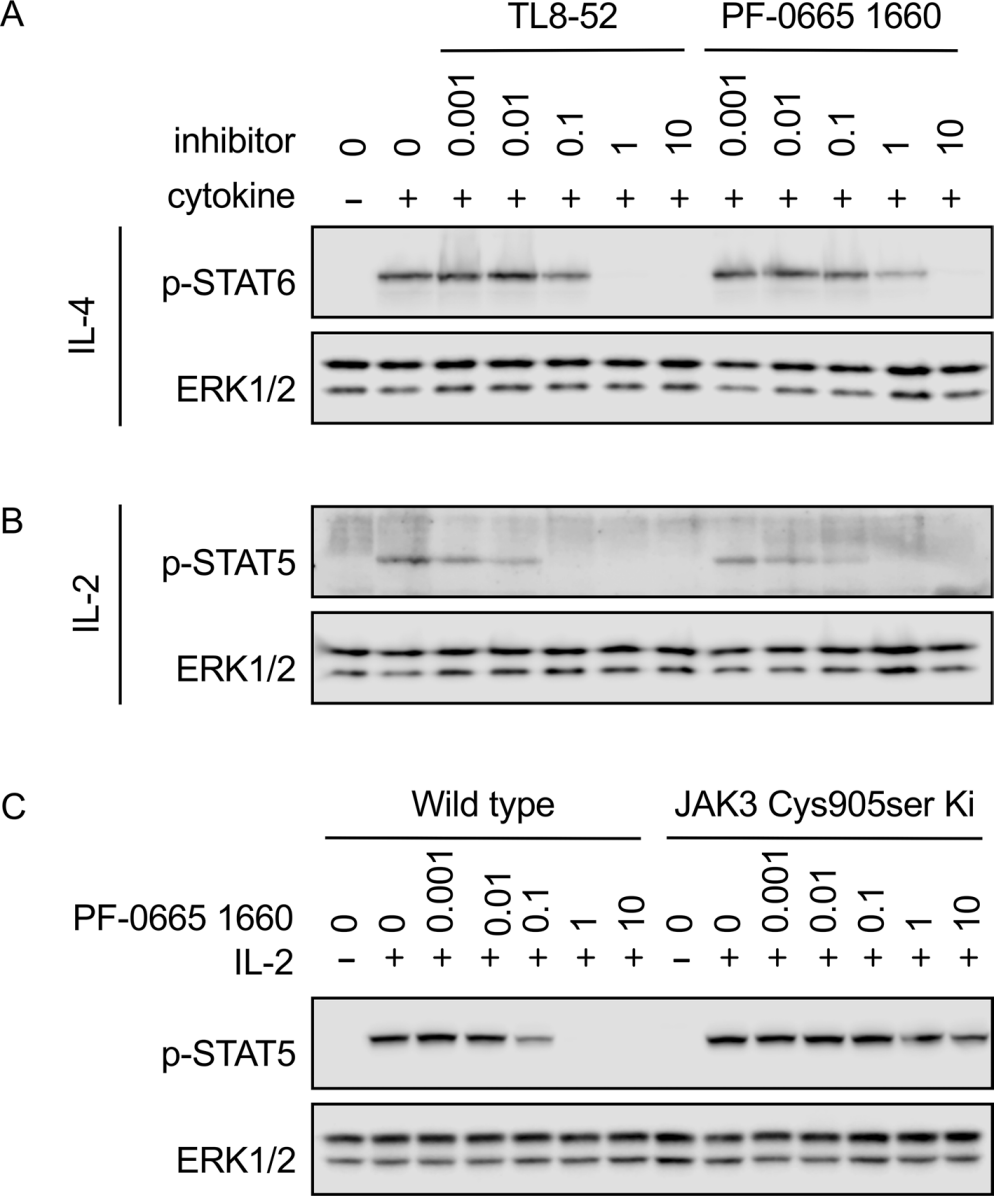
Effect of TL8-52 and PF-06651600 on cytokine signaling in spleenocytes and T cells. (**A**) Single cell suspensions were prepared from the spleens of wild type mice. Following red blood cell lysis, cells were incubated for 1 h in the presence of the indicated concentrations in μM of TL8-52 or PF-06651600. Cells were then stimulated with 10 ng/ml IL-4 for 30 min and the levels of phosphorylated STAT6 determined by immunoblotting. (**B**) As (**A**) except cells were stimulated with 10 ng/ml IL-2 and the levels of phosphorylated STAT5 determined. (**C**) CD8 T cells were expanded from the lymph nodes of wild type and JAK3 Cys905Ser knockin mice. Cells were rested in the absence of IL-2 for 60 min then incubated with the indicated concentrations of PF-06651600 for a further 30 min before stimulation with 10 ng/ml of IL-2 for 30 min. The levels of phosphorylated STAT5 and total ERK1/2 were then determined.

The above data suggest that TL6-144, TL8-52, PF-06651600 and FM381 block IL-4 signaling via inhibition of JAK3 and not JAK1. If this was the case, then they should only block STAT phosphorylation downstream of JAK3 dependent cytokines. Like IL-4, IL-2 requires JAK1 and JAK3 for signaling. Consistent with the results in macrophages both PF-06651600 and TL8-52 were able to block IL-4 induced STAT6 phosphorylation in spleenocytes (Fig. 5A). A similar concentration of either TL8-52 or PF-06651600 was also able to block IL-2 induced STAT5 phosphorylation (Fig. 5B). In line with this, PF-06651600 was also able to block IL-2 induced STAT5 phosphorylation in isolated T cells (Fig. 5C). In contrast to wild type T cells, PF-06651600 did not block IL-2 induced STAT5 phosphorylation in T cells from JAK3 Cys905Ser knockin mice.

**Figure 6:**
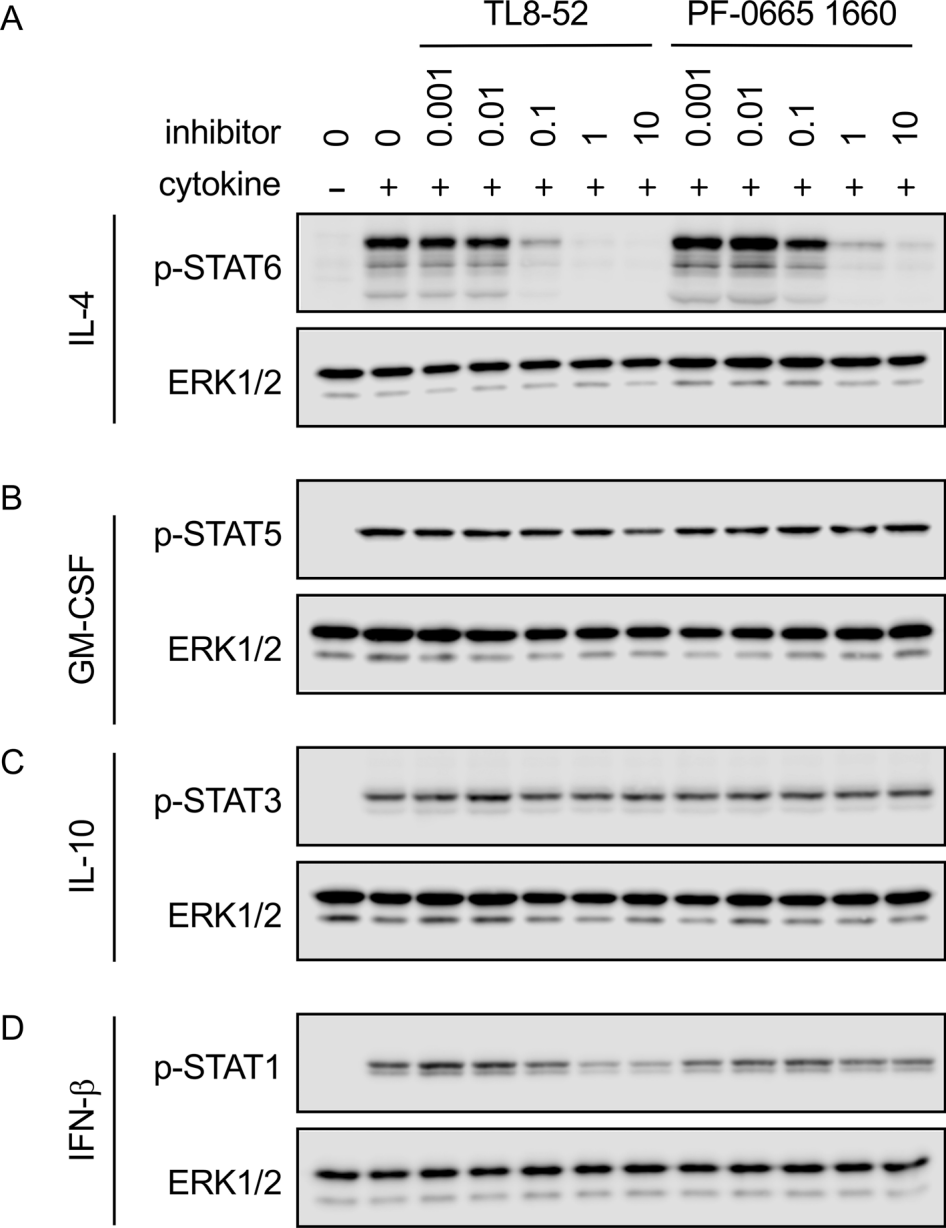
Effect of TL8-52 and PF-06651600 on cytokine signaling in macrophages. (**A**) BMDMs were isolated from wild type mice. Cells were incubated with the indicated inhibitors in μM for 2 h before stimulation with 10 ng/ml IL-4 for 30 min. The levels of phosphorylated STAT6 were then determined by immunoblotting. (**B**) As (**A**) except cells were stimulated 10 ng/ml GM-CSF and the levels of phosphorylated STAT5 determined. (**C**) As (**A**) except cells were stimulated 10 ng/ml IL-10 and the levels of phosphorylated STAT3 determined. (**D**) As (**A**) except cells were stimulated 10 ng/ml IFNβ and the levels of phosphorylated STAT1 determined. For **A**–**D**, levels of ERK1/2 were determined as a loading control.

In addition, the effects on JAK3 independent cytokines were also examined. GM-CSF signals via JAK2 while IL-10 and IFNβ signal via JAK1 and Tyk2. We have previously shown that TL6-144 does not inhibit GM-CSF, IFNβ or IL-10 signaling in macrophages at a concentration of 5 μM^25^. Neither TL8-52 or PF-06651600 inhibited GM-CSF induced STAT5 phosphorylation at 10 μM, a concentration sufficient to block IL-4 signaling (Fig. 6A,B). TL8-52 or PF-0665160 also had no effect on IL-10 signaling (Fig. 6C). PF-06651600 did not block IFNβ induced STAT1 phosphorylation at 10 μM (Fig. 6D). Some inhibition of STAT1 phosphorylation was seen with TL8-52, however the concentration required to inhibit STAT1 phosphorylation in response to IFNβ was higher than that required to block IL-4 induced STAT6 phosphorylation (Fig. 6D).

**Table 1:** In vitro IC50 values for the covalent JAK3 inhibitors used.

	JAK3	JAK1	JAK2	TYK2	References
TL6-144	4.8 (10)	896 (75)	1050 (25)	> 10,000 (25)	^25^
TL8-52	1.3 (10)	24.4 (75)	17.6 (25)	320 (25)	^25^
PF-06651600	0.35 (4)	> 10,000 (40)	> 10,000 (4)	> 10,000 (12)	^24^
FM-381	0.127 (10)	52 (10)	346 (10)	459 (10)	^23^

Published IC50 values (in nM) for the covalent JAK3 inhibitors used in this study are shown. ATP concentrations in μM used in the assays are shown in brackets.

## Discussion

We report here the generation of mice with a Cys905 to serine knockin mutation in the endogenous JAK3 gene. This mutation does not block catalytic activity but does affect the ability of covalent JAK3 inhibitors to block JAK3 function. Importantly, the knockin mice do not show the issues in T and B cell development that have been reported in JAK3 knockout mice, indicating the Cys905Ser mutation does not interfere with JAK3 function in vivo (Fig. 2). Consistent with this, the phosphorylation of STAT6 in response to IL-4, a cytokine that signals via JAK1 and JAK3 was unaffected by the Cys905Ser mutation (Fig. 3).

JAKs have become important targets in autoimmune disease, however the ideal profile of a JAK inhibitor to treat autoimmunity is not fully understood. JAK3 was initially suggested as an ideal target as its expression is restricted to the immune system and loss of JAK3 function results in SCID. JAK3 binds the common gamma chain (γ_c_) receptor, which is required for the action of IL-4, IL-5, IL-7, IL-15 and IL-21. For each of these cytokines, there is a cytokine specific β receptor chain that recruits JAK1, resulting in both JAK1 and JAK3 being required for signalling downstream of these cytokines. In addition to its role in γ_c_ cytokine receptor signalling, JAK1 is also required for signalling downstream of other cytokine receptors including interferon receptors and gp130 containing receptors. Thus JAK1 inhibitors would block a broader range of cytokines that JAK3 inhibitors.

While JAK3 is required for signalling by γ_c_ dependent cytokines it has been less clear if the kinase activity of JAK3 is critical or if the main role of JAK3 is as a scaffold protein or in transporting the (γ_c_) receptor to the plasma membrane^38^. In the context of γ_c_ cytokine receptors, JAK1 kinase activity has been suggested to have a dominant role over JAK3. Using a cell line derived from a JAK3 deficient patient it was found the JAK3 was required for IL-2 induced STAT5 phosphorylation but was not essential for IL-4 induced STAT6 phosphorylation^39^. Initial studies on inhibitors selective for JAK3 over JAK1 in in vitro kinase assays found that this selectively was lost in cellular assays^40^. This change in selectivity was suggested to be due to JAK3 having a lower Km for ATP than JAK1 in in vitro kinase assays^41^. As in vitro assays are normally performed with ATP values close to the Km, it is possible that for ATP competitive JAK3 inhibitors selectivity over JAK1 is reduced at the high ATP concentrations found in the cells. To address this Hann et al.reconstituted IL-2 signalling in U4C cells by over expressing the IL-2 receptor along with combinations of JAK1 and JAK3 or gatekeeper mutations in these kinases that make the kinase sensitive to inhibition by 1NM-PP1. Using this approach they found that JAK1 kinase activity but not JAK3 kinase activity was essential for IL-2 induced STAT5 phosphorylation^42,43^. In contrast another study showed that retroviral transfection of a JAK3 Cys905Ser mutant could prevent the inhibition of IL-2 induced STAT5 phosphorylation by a covalent JAK3 inhibitor, arguing for an important role for JAK3 kinase activity^44^. These studies however make use of overexpression and it is possible that the selective requirement for JAK1 kinase activity may not be maintained at endogenous levels of receptor and JAK expression.

A highly selective JAK3 inhibitor would potentially resolve some of these issues, however obtaining the required selectivity for JAK3 over JAK1 with a purely ATP competitive inhibitor has not been straightforward. Compounds that target the cysteine present in the JAK3 binding pocket have been described as highly selective JAK3 inhibitors *in vitro*^23–32^. These compounds however act via a two-step mechanism–an initial reversible ATP competitive binding to JAK3 followed by the covalent modification of Cys905. These compounds therefore retain the potential to act as ATP competitive kinase inhibitors in the absence of covalent modification, albeit at a much reduced potency. Mutation of Cys905 in the JAK3 gene however provides a way of confirming if these compounds act via JAK3 or an off-target mechanism. Using cells from a JAK3 Cys905Ser knockin mouse we show that the IC50 values for IL-4 induced STAT6 phosphorylation is increased when these compounds cannot covalently modify JAK3 (Fig. 3). The magnitude of this increase varied between different inhibitors, which may reflect their ability to bind to the JAK3 ATP binding pocket in an ATP competitive manner or an off target activity against JAK1. Distinguishing between these two in cells is not straight forward. Extrapolating the in vitro IC50 values for the inhibitors to cellular IC50 values is problematic, as cellular ATP concentrations are much higher than those used for in vitro assays. Furthermore the in vitro assays use an isolated kinase domain rather than the full length protein containing the kinase and pseudo-kinase domains. Of note however, TL8-52 which had the lowest selectivity for JAK3 over JAK1 in vitro showed only a moderate increase in the IC50 for IL-4 inducted STAT6 phosphorylation and also showed some inhibition of IFNβ induced STAT1 phosphorylation at 10 μM. As type 1 interferon requires JAK1 to induce STAT1 phosphorylation^16^, this could indicate that in cells TL8-52 may hit both the JAK1 and the JAK3 Cys905Ser at 5–10 μM. In contrast to its effects on IFNβ signalling, TL8-52 did not block IL-10 induced STAT3 phosphorylation, which also requires JAK1^16,45^.

TL6-144 and PF-06651600 were the most selective of the compounds used for JAK3 based on the in vitro assays (Table 1) and did not block STAT phosphorylation by the JAK3 independent cytokines IL-10, IFNβ and GM-CSF at 10 μM (Fig. 6)^25^. Furthermore, they both had a much lower IC50 for IL-4 induced STAT6 phosphorylation in wild type relative to JAK3 Cys905Ser macrophages. Together this would suggest that at concentrations of 5 to 10 μM in wild type cells they are active against JAK3 but not other JAK isoforms. Consistent with these compounds inhibiting IL-4 induced STAT6 phosphorylation via a covalent inhibition of JAK3, their ability to retain inhibition in washout experiments was dependent on the presence of Cys905 in JAK3. Together these would indicate that inhibiting JAK3 kinase activity is sufficient to block STAT phosphorylation downstream of γ_c_ dependent cytokines. Consistent with this, PF-06651600 has progressed into phase II clinical trials for a number of conditions, including ulcerative colitis, Crohn’s disease, vitiligo, alopecia areata and rheumatoid arthritis, with promising results recently being reported in the phase II trial for rheumatoid arthritis^46^. PF-06651600 has also been reported to inhibit some TEC family kinases, and so it is possible that its therapeutic effects could be due to combined inhibition of JAK3 and TEC kinases^46^.

In summary, we report here a JAK3 Cys905Ser knockin mouse in which JAK3 shows reduced inhibition by covalent JAK3 inhibitors. This would provide a model for studying JAK3 function in vivo, and evaluation of the potential on target and off target effects of these covalent JAK3 inhibitors in vivo. It is possible that mutation of the equivalent serine in other JAK isoforms could be used to engineer sensitivity of other JAKs to covalent inhibitors and therefore expanding this approach to other JAK isoforms may provide additional tools to study the catalytic roles of other JAK family members.

## Methods

### Materials

TL6-144 and TL8-52 were generated in house as described previously and correspond to compound 9 and 21 in^25^. PF-06651600^24^ and Ruxolitinib^47^ were obtained from Sigma and Selleck Chemicals respectively. FM-381^23^ was kindly provided by Stefan Laufer (University of Tübingen). Published in vitro IC50 values for the JAK3 inhibitors used is given in Table 1.

### Cloning and expression JAK3 Kinase domains

The coding region for Jak3 I781-S1124 (NP_000206.2) was amplified using oligonucleotides adding Bam HI and Not I restriction sites at the 5′ and 3′ ends of the cDNA, respectively. The amplified product was cloned into the holding vector pSC-B (Stratagene), sequence-checked and then subcloned into a pFastbacDual plasmid downstream of the GST and PreScission protease sequence using Bam HI and Not I. All PCR reactions were carried out using KOD Hot Start DNA polymerase (Merck). The C909S mutation was introduced by PCR mutagenesis using KOD Hot Start DNA polymerase and complementary oligos containing the mutation.

Constructs encoding JAK3 or the C909S mutant with an N-terminal Glutathione-S-transferase (GST) tag were used to generate recombinant baculovirus using the Bac-to-Bac system (Invitrogen) following the manufacturer's protocol. The resulting baculovirus were used to infect *Spodoptera frugiperda* 21 cells, the infected cells were grown at 27 °C, harvested 48 h post-infection and the GST-tagged JAK3 proteins purified on GSH Agarose (Expedeon) and dialysed into 50 mM Tris–HCl pH 7.5, 0.1 mM EGTA, 150 mM NaCl, 0.1% 2-mercaptoethanol, 270 mM Sucrose, 0.03% Brij-35 and stored at − 80 °C. More detailed information for the constructs encoding JAK3 [DU25657] or the C909S mutant [DU50565] as well as recombination proteins generated for this study can be found at https://mrcppureagents.dundee.ac.uk/.

### In vitro* kinase assays*

For the in vitro kinase assay, JAK3 (5–20 mU diluted in 50 mM Tris pH 7.5, 0.1 mM EGTA, 0.05% β-mercaptoethanol, 1 mg/ml BSA) was assayed against PDKtide (KTFCGTPEYLAPEVRREPRILSEEEQEMFRDFDYIADWC) in a final volume of 25.5 µl containing 50 mM Tris pH 7.5, 0.1 mM EGTA, 0.05% β-mercaptoethanol, 100 µM substrate peptide, 10 mM magnesium acetate and 0.02 mM [33P-γ-ATP] (50–1000 cpm/pmole) and incubated for 30 min at room temperature. Assays were started by the addition of the ATP and stopped by addition of 5 µl of 0.5 M (3%) orthophosphoric acid and then harvested onto P81 Unifilter plates with a wash buffer of 50 mM orthophosphoric acid. To determine the Km for ATP, assays were run using 1, 5, 10, 20, 50, 100, 200, 400, 500 μM ATP and Km derived by non-linear regression fitting to the Michaelis–Menten equation v = Vmax[S]/(Km + [S]). IC50 values for inhibitors via non-linear regression using a parameter model: Y = Bottom + (Top–Bottom)/(1 + (IC50/X)^HillSlope). Non-linear regression was carried out in Prism (version 8). In vitro selectivity screen was carried out as described previously^48^.

### Animals

Mice with a Cys905 to Serine mutation in the kinase domain of JAK3 were generated via CRISPR/Cas9 technology. Cys905 is encoded in exon 20 of the JAK3 gene. Briefly, a guide RNA sequence (GGCGCTGCAGGAAGTCTCGCAGG) was selected to overlap with Cys905 in exon 20. Analysis of the mouse genome (GRCm38/mm10 assembly) suggested that this guide had at least 4 mismatches’ in the closest off target sequences. An oligo was also designed for homology mediated repair of exon 20 that incorporated the mutation of Cys905 to serine as well as further silent mutation to introduce Hind III and Eco R1 sites (GGCTGCCTCCAG to GGAAGCTTAAGG). Cas9 protein, gRNA and the repair oligo were microinjected into C57Bl/6NTac zygotes. Founders were screened by PCR across exons 20 and 21 followed by restriction analysis and sequencing to identify animals with a Cys905Ser knockin mutation. F0 animals were then crossed to wild type C67Bl/6 mice and F1 offspring screened for the presence of the Cys905Ser mutation in JAK3. Heterozygous JAK3 knockin mice were then crossed to generate homozygous JAK3 Cys905Ser knockin mice. Routine genotyping was carried out by PCR of ear biopsies using the primers TGAACAAGGTCGTTAACTCCC and TCTGGAGTCTTGGTCTTGTACC followed by digestion of the product with Hind III. This resulted in a 504 bp band from a wild type allele or 354 and 150 bp bands from a knockin allele.

Nonbreeding mice were housed in same-sex groups, in individually ventilated sterile cages and were given free access to food (R&M1 SDS, Special Diets Services) and water. Animals were maintained in rooms with controlled 12 h/12 h light/dark cycle, 21 °C temperature, and relative humidity of 45–65%. All the work was performed under a UK Home Office project license in accordance with UK and EU regulations and ethical approval obtained from the University of Dundee Ethical Review Committee and compliant with ARRIVE guidelines (http://www.nc3rs.org.uk/page.asp?id=1357).

### Flow cytometry

For flow cytometry, samples were treated as described previously^49^. Briefly, thymi, spleens and lymph nodes were removed and disaggregated in RPMI media. Whole blood and spleens were treated with RBC lysis buffer (Sigma). Single cell suspensions, diluted in PBS, were used to analyse total cell counts on BD FACSVerse (BD Biosciences). For phenotyping, cells were washed with FACS buffer (1% BSA in PBS) and Fcγ receptors were blocked with Mouse BD Fc Block (BD Biosciences) for 15 min at 4 °C. Cell surface markers on thymocytes were then stained with anti-Thy1.2-APC, anti-CD4-PE, anti-CD8-BV421 and anti-TCRβ-FITC in FACS buffer for 20 min at 4 °C. Cells from the spleen and lymph nodes were stained with anti- TCRβ-FITC, anti-CD4-PerCP-Cy5.5, and anti-CD8-APC to identify T cells and anti-CD19-APC, anti-IgM-FITC and anti-IgD-PE to identify B cells. Blood cells were stained with anti-CD3-FITC and anti-CD19-APC. Details of the antibodies used are in supplemental table 2. Stained cells were washed and resuspended in FACS buffer and acquired on BD FACSCanto II (BD Biosciences) using FACSDiva software. Analysis was done on FlowJo.

### Macrophage culture

Bone marrow derived macrophages (BMDMs) were cultured as described^50^. Briefly, bone marrow was flushed in PBS from the femurs and tibias of mice. Cells were cultured on bacterial grade plastic for 7 days at 37 °C, 5% CO_2_. Cells were then detached using Versene (Gibco), re-plated on tissue culture plastic in BMDM media (in DMEM supplemented with 10% heat inactivated FBS (Labtech), 2 mM L-glutamine, 100 U/ml penicillin G, 100 μg/ml streptomycin and 0.25 μg/ml amphotericin, 5 ng/ml M-CSF) and used within 48 h. Where indicated cells were stimulated with 10 ng/ml IL-4, 10 ng/ml GM-CSF, 10 ng/ml IL-10 or 10 ng/ml IFNβ. All cytokines were from PeproTech.

### T cell culture

Lymph Nodes were harvested from the mice and a single cell suspension in PBS made by passing the tissue through a 10 μm strainer. The cells were counted and seeded at density of 10^6^ cells/ml in RPMI supplemented with10% heat inactivated FBS (Labtech), 2 mM L-glutamine, 100 U/ml penicillin G, 100 μg/ml streptomycin, 0.25 μg/ml amphotericin and 50 μM β-mercaptoethanol and stimulated with 1 μg/ml anti-CD28 (cat:16-0281-82; clone:37.51 Thermo Fisher), 1 μg/ml anti CD3e (cat:16-0031-85, Clone:145-2C11 Thermo Fisher), 20 ng/ml of IL-2 and 1 ng/ml of IL-12. The cells were cultured for 36 h and then the cell suspension was diluted 5 times with RPMI media containing 20 ng/ml of IL-2. The cells were seeded and maintained at 300,000 cells/ml and split every consequent day.

### Immunoblotting

Cells were lysed directly into SDS sample buffer and aliquots run on 10% polyacrylamide gels using standard methods^51^. Proteins were transferred onto nitrocellulose membranes, and specific proteins were detected by immunoblotting. Antibodies against tyrosine phosphorylated STATs were from Cell Signaling Technology (phospho-STAT1 #9167, phospho-STAT3 #9131, phospho-STAT5 #4322 and phospho-STAT6 #9361). The total ERK1/2 antibody used as a loading control was also from cell Signaling Technology (#4695). HRP-conjugated secondary antibodies were obtained from Pierce. Bands were detected using Clarity ECL reagents from BioRad and imaged on a Licor Odyssey Fc system. Quantification was carried out using Licor Image Studio software. After correction for the ERK1/2 levels to account for loading differences, percentage inhibition was calculated relative to the IL-4 stimulated condition. IC50 values were calculated using the same methods as for the in vitro IC50 values.

## Supplementary Information

Supplementary Information
