# Supplementary material for: Generation of a chemical genetic model for JAK3
Source: Sci Rep. 2021 May 12;11:10093. doi: 10.1038/s41598-021-89356-4 (PMC8115619; doi:10.1038/s41598-021-89356-4)

Supplementary information

Generation of a chemical genetic model for JAK3.

Judit Remenyi[1], Rangeetha Jayaprakash Naik[1], Jinhua Wang[2], Momchil Razsolkov[1], Alyssa Verano[2], Quan Cai[2], Li Tan[2], Rachel Toth[3], Samantha Raggett[3], Carla Baillie[3], Ryan Traynor[3], C. James Hastie[3], Nathanael S. Gray[2] and J. Simon C. Arthur[1*].

1: Division of Cell Signalling and Immunology, School of Life Sciences, Wellcome Trust Building, University of Dundee, Dundee, DD1 5EH, UK.
2: Department of Cancer Biology, Dana Farber Cancer Institute, Department of Biological Chemistry and Molecular Pharmacology, Harvard Medical School, USA.
3: MRC PPU Reagents and Services, School of Life Sciences, University of Dundee, Dundee, DD1 5EH, UK.

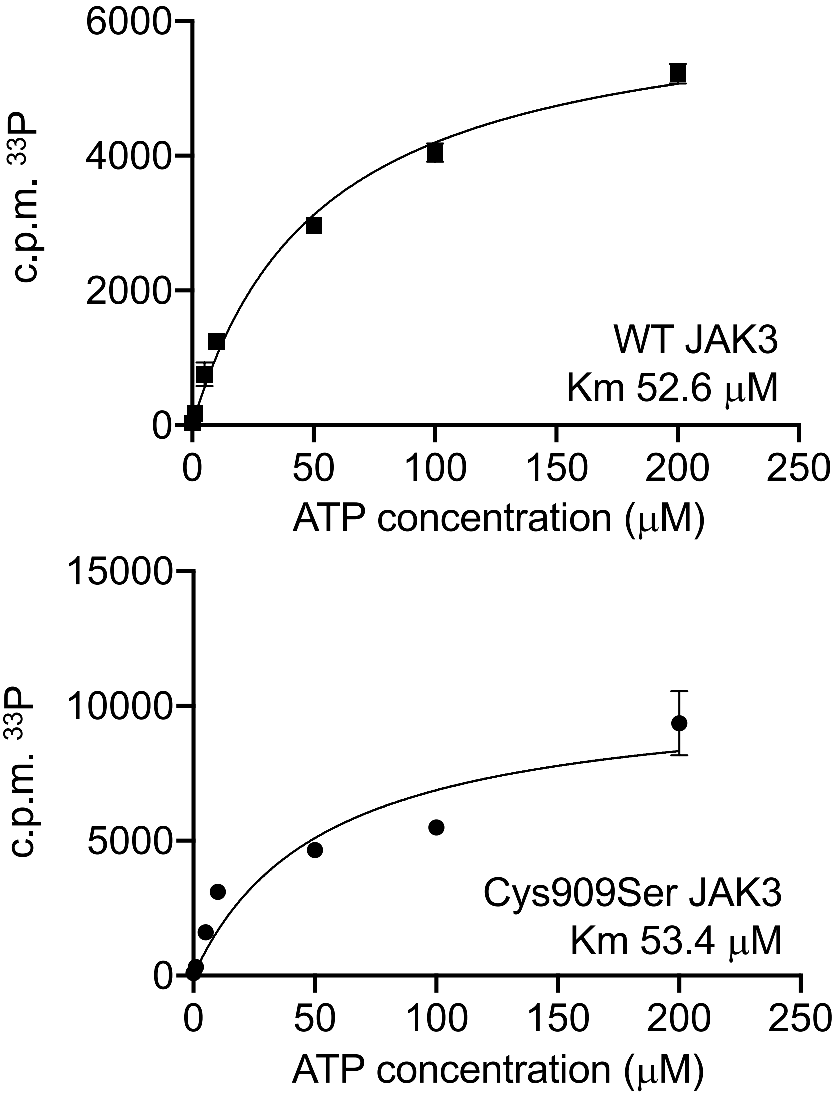

Supplementary Figure 1. Km for ATP of isolated JAK3 kinase domains.
Recombinant wild type and Cys909Ser JAK3 kinase domains were expressed and purified from insect cells as described in the methods. Kinase assays for the purified kinase domains were run using 1, 5, 10, 20, 50, 100, 200, 400, 500 μM ATP. The Km was derived by non-linear regression fitting to the Michaelis-Menten equation v=Vmax[S]/(Km+[S]). IC50 values for inhibitors via non linear regression using a parameter model: Y=Bottom + (Top-Bottom)/(1+(IC50/X)^HillSlope). Non linear regression was carried out in Prism (version 8). Graphs show mean and standard deviation of 2 replicates and the line indicated shows the fitted curve.

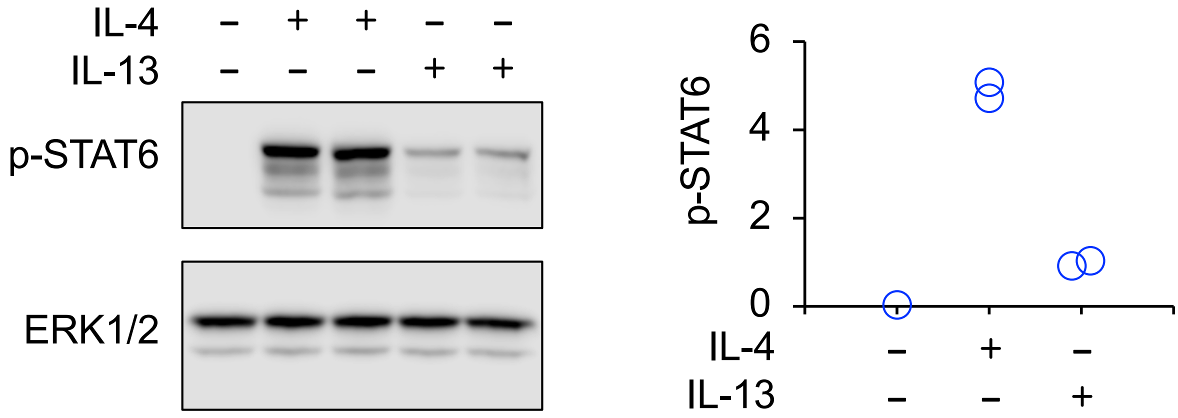

Supplementary Figure 2. Comparison of IL-4 and IL-13 induced STAT6 phosphorylation.
Macrophages were stimulated for 30min with either 10ng/ml IL-4 or 10ng/ml IL-13. Cells were then lysed and the levels of phopsho-STAT6 and total ERK1/2 determined by immunoblotting. Gel images are shown on the left and quantification of the phoshpho-STAT6 signal normalised to the levels of total ERK1/2 on the right.

Supplementary table 1: Selectivity screening data for TL6-144 and TL8-52

TL6-144 and TL87-52 were screened at 1 μM against a panel of kinases in vitro as described in the methods. Data shows mean and standard deviation of the activity remaining in the presence of the inhibitor

Kinase	TL6-144	stdev	TL8-52	stdev
ABL	87	12	89	6
AMPK	91	5	80	6
ASK1	108	3	93	4
Aurora A	94	8	13	1
Aurora B	81	0	13	2
BRK	94	11	94	36
BRSK1	103	16	86	9
BRSK2	90	7	87	19
BTK	37	7	44	2
CAMK1	68	4	97	0
CAMKKb	6	1	22	2
CDK2-Cyclin A	87	4	84	10
CDK9-Cyclin T1	95	0	80	3
CHK1	93	13	91	12
CHK2	38	1	75	4
CK1γ2	82	3	96	10
CK1δ	83	5	110	2
CK2	103	5	103	10
CLK2	62	6	29	3
CSK	113	6	73	8
DAPK1	94	7	100	6
DDR2	83	1	76	9
DYRK1A	90	10	100	7
DYRK2	92	14	87	0
DYRK3	95	3	95	7
EF2K	95	0	105	0
EIF2AK3	98	5	90	9
EPH-A2	72	3	87	6
EPH-A4	104	10	99	20
EPH-B1	85	5	75	0
EPH-B2	96	8	81	22
EPH-B3	77	0	73	8
EPH-B4	83	6	104	9
ERK1	94	4	94	17
ERK2	106	18	97	4
ERK5	87	6	63	2
ERK8	90	8	65	10
FGF-R1	44	13	49	14
GCK	92	10	21	2
GSK3b	87	5	118	4
HER4	9	0	31	6
HIPK1	79	8	96	6
HIPK2	87	15	97	4
HIPK3	91	6	107	3
IGF-1R	5	1	24	9
IKKb	100	15	104	8
IKKe	100	6	34	4
IR	11	1	15	0
IRAK1	81	5	54	7
IRAK4	85	9	77	4
IRR	56	4	75	7
JNK1	83	5	104	21
JNK2	98	9	95	2
JNK3	87	4	69	4
Lck	77	2	73	10
LKB1	91	11	132	19
MAP4K3	83	9	55	1
MAP4K5	83	8	63	10
MAPKAP-K2	103	5	97	3
MAPKAP-K3	119	3	62	13
MARK1	87	2	44	8
MARK2	84	4	37	12
MARK3	48	4	21	0
MARK4	76	2	28	2
MEKK1	95	14	103	13
MELK	69	3	66	12
MINK1	80	4	80	10
MKK1	79	2	84	19
MKK2	93	4	63	5
MKK6	104	4	105	3
MLK1	20	4	3	1
MLK3	31	0	3	1
MNK1	105	6	112	14
MNK2	70	2	66	4
MPSK1	91	10	98	9
MSK1	86	9	54	9
MST2	83	14	48	0
MST3	99	5	97	17
MST4	107	27	96	12
NEK2a	96	11	135	44
NEK6	97	11	103	4
NUAK1	4	0	3	1
OSR1	47	10	98	8
p38a MAPK	96	5	119	5
p38b MAPK	93	3	96	5
p38d MAPK	94	1	93	5
p38g MAPK	88	4	93	2
PAK2	81	10	75	14
PAK4	99	3	85	5
PAK5	94	7	90	22
PAK6	77	10	99	16
PDGFRA	67	5	35	4
PDK1	101	0	86	8
PHK	40	12	78	3
PIM1	95	5	111	13
PIM2	97	2	100	6
PIM3	100	5	90	13
PINK	107	1	110	2
PKA	104	20	74	6
PKBa	105	8	100	4
PKBb	107	21	107	6
PKCa	81	6	91	14
PKCz	88	6	98	7
PKCγ	111	6	109	5
PKD1	46	9	86	9
PLK1	107	6	114	10
PRAK	93	11	113	22
PRK2	78	0	52	4
RIPK2	92	9	71	13
ROCK 2	83	3	93	25
RSK1	61	2	60	0
RSK2	73	10	53	8
S6K1	90	2	87	13
SGK1	100	7	89	13
SIK2	58	2	31	3
SIK3	89	16	59	6
SmMLCK	62	16	98	20
Src	74	5	53	0
SRPK1	91	9	97	4
STK33	81	5	94	9
SYK	107	7	63	8
TAK1	77	5	39	4
TAO1	99	1	91	18
TBK1	78	3	11	1
TESK1	99	6	91	6
TGFBR1	83	8	104	15
TIE2	87	5	116	16
TLK1	102	8	82	9
TrkA	59	5	23	3
TSSK1	34	0	57	3
TTBK1	80	2	100	10
TTBK2	94	6	106	8
TTK	96	5	49	10
ULK1	65	12	48	2
ULK2	63	11	92	1
VEG-FR	39	3	6	1
WNK1	81	21	112	10
YES1	89	8	47	14
ZAP70	86	5	77	32

Supplementary table 2: Flow cytometry antibodies

Antibody	Clone	Supplier
APC-anti Thy1.2	53-2.1	Biolegend
PE-anti-CD4	GK1.5	BD
Bv421-anti CD8a	53-6.7	Biolegend
FITC-anti TCRb	H57-597	Biolegend
PerCP-Cy5.5 anti-CD4	GK1.5	Biolegend
APC-anti CD8a	53-6.7	Biolegend
APC-anti CD19	6D5	Biolegend
FITC-anti IgM	1B4B1	Southern Biotech
PE-anti IgD	11-26c.2a	BD

Supplementary data. Scans of immunoblots used in the generation of figures chemiluminescent

All blots were imaged using a Licor Odyssey FC imaging system using a combination of the chemiluminescent (to detect antibody signals) and 700nM (to image pre-stained molecular weight markers) channels and processed using Image Studio software. Images below show an overlay of the chemiluminescent (red) and 700nM (blue). Images used if the main figures show the chemiluminescent channel only.

Blots used to generate figure 3

TL6-144, phopho-STAT6

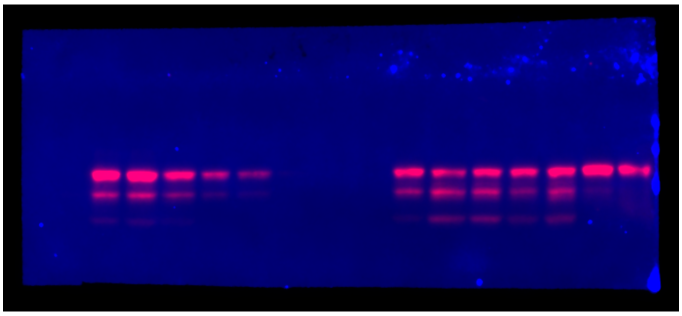

TL6-144, total ERK1/2

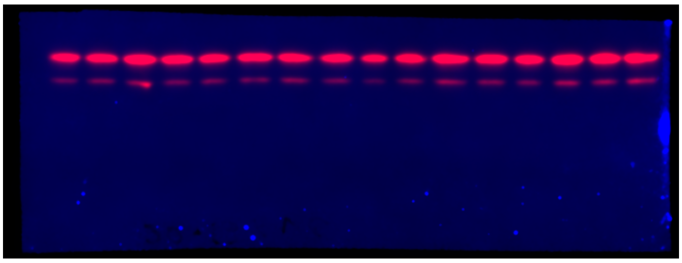

TL8-52, phopho-STAT6

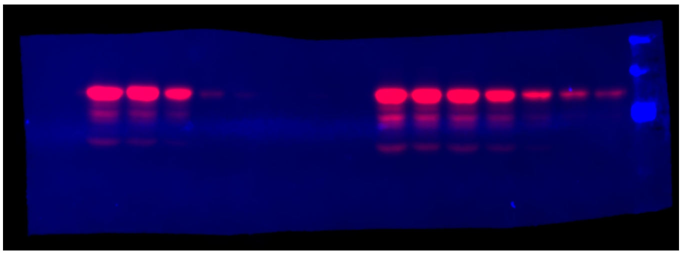

TL8-52, total ERK1/2

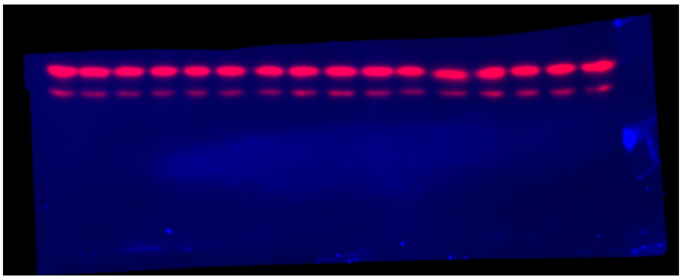

PF06651600, phopho-STAT6

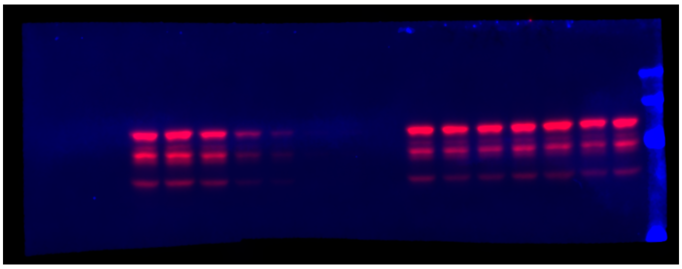

PF06651600, total ERK1/2

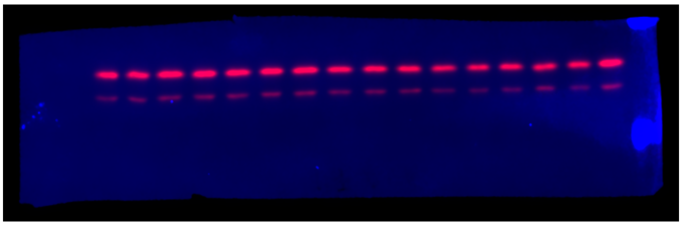

FM381, phopho-STAT6

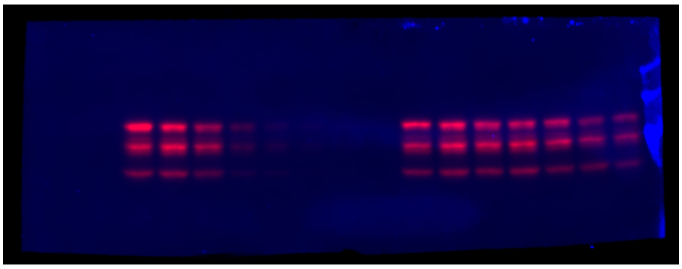

FM-381, total ERK1/2

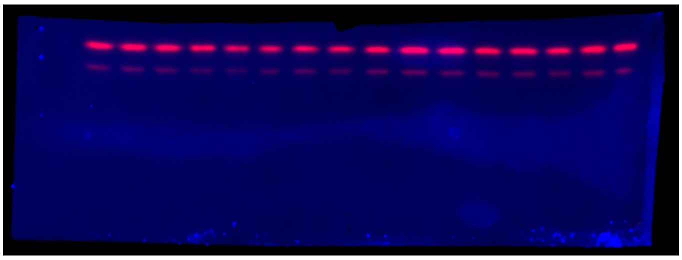

Blots used to generate figure 4

Wild type, TL6-144, phopho-STAT6

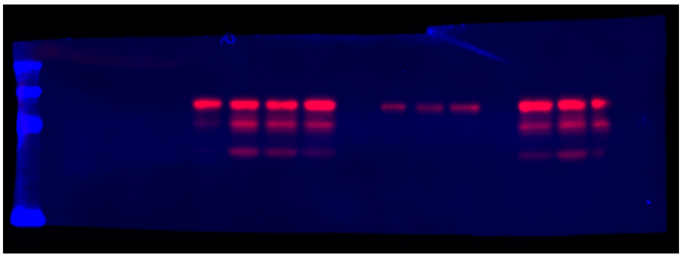

Cys905Ser Ki, TL6-144, phopho-STAT6

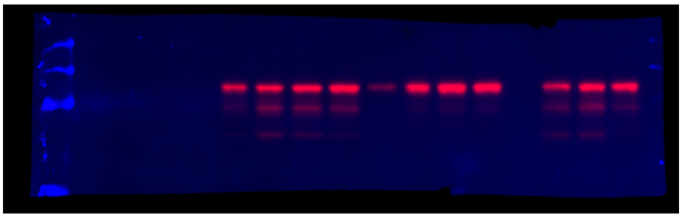

Wild type, TL6-144, total-ERK1/2

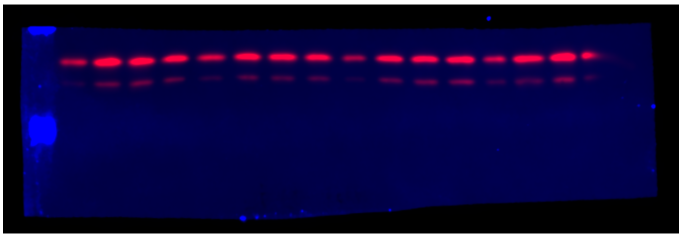

Cys905Ser Ki, TL6-144, total-ERK1/2

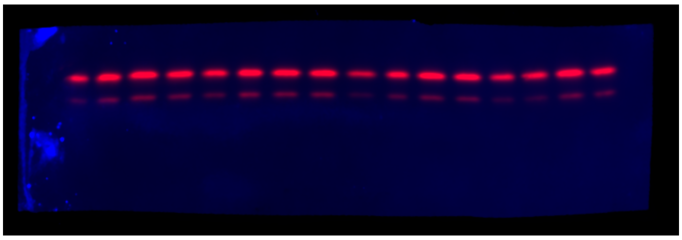

Wild type, PF-06651600, phopho-STAT6, 0 – 2h

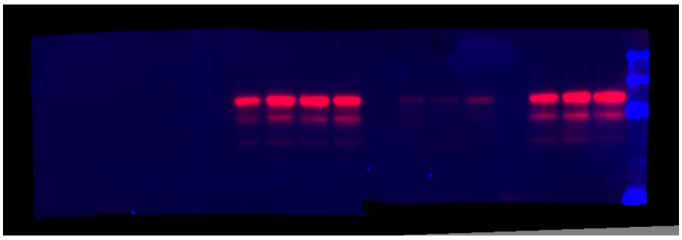

Cys905Ser Ki, PF-06651600, phopho-STAT6, 0 – 2h

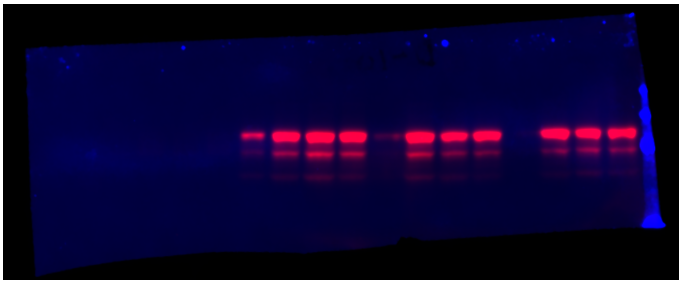

Wild type, PF-06651600, total-ERK1/2, 0 – 2h

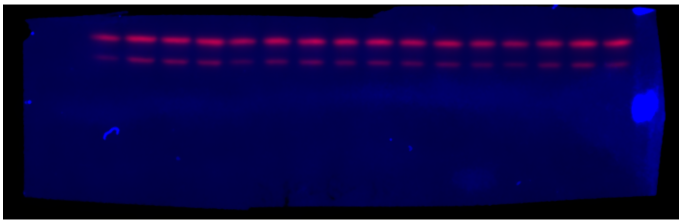

Cys905Ser Ki, PF-06651600, total-ERK1/2, 0 -2h

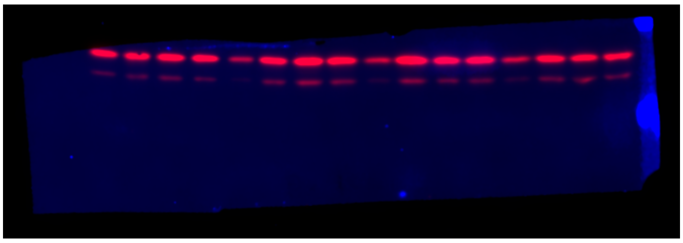

Wild type, PF-06651600, phopho-STAT6, 0 – 24h

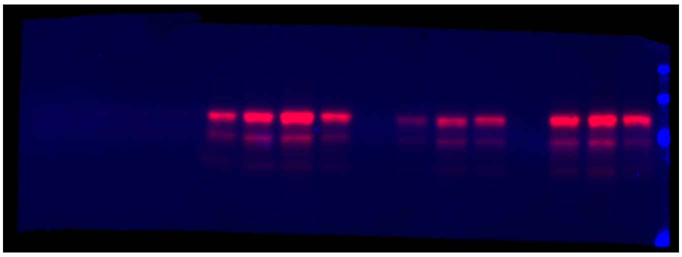

Cys905Ser Ki, PF-06651600, phopho-STAT6, 0 – 24h

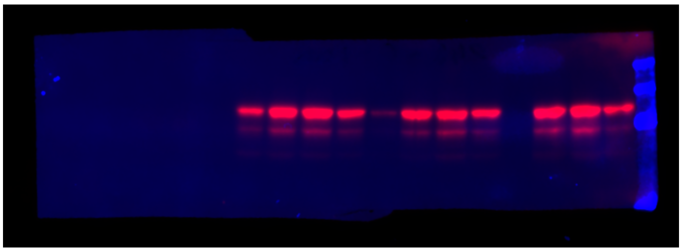

Wild type, PF-06651600, total-ERK1/2, 0 – 24h

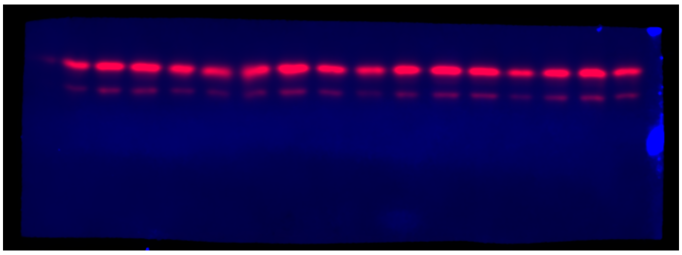

Cys905Ser Ki, PF-06651600, total-ERK1/2, 0 -24h

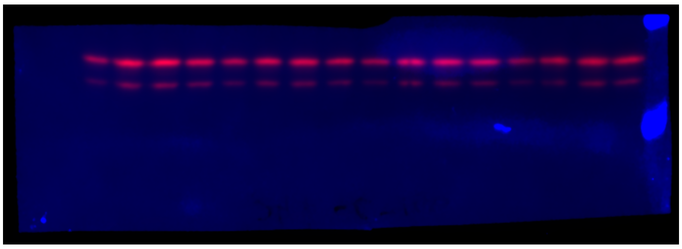

Blots used to generate figure 5

A Spleenocytes, IL-4, phospho-STAT6

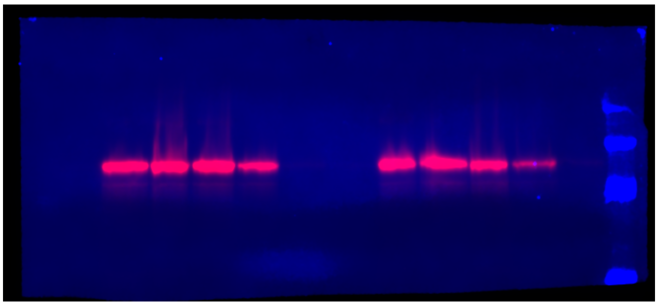

Spleenocytes IL-4, total ERK1/2

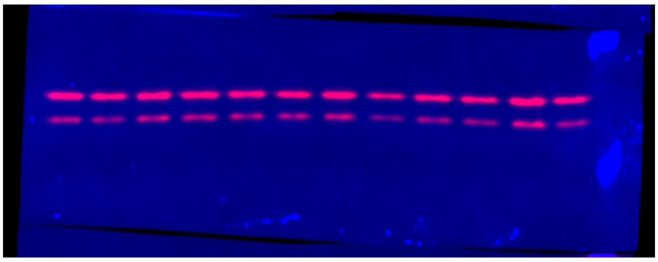

B Spleenocytes, IL-2, phospho-STAT5

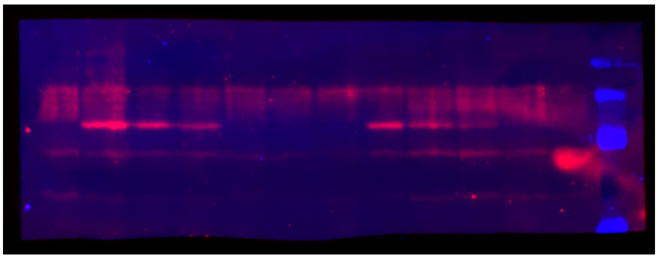

Spleenocytes, IL-2, total ERK1/2

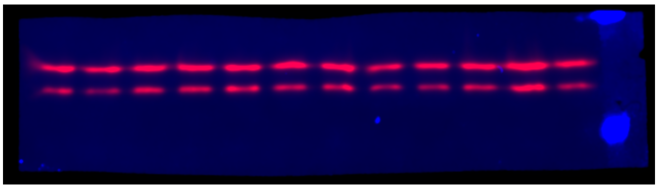

B T cells, IL-2, phospho-STAT5

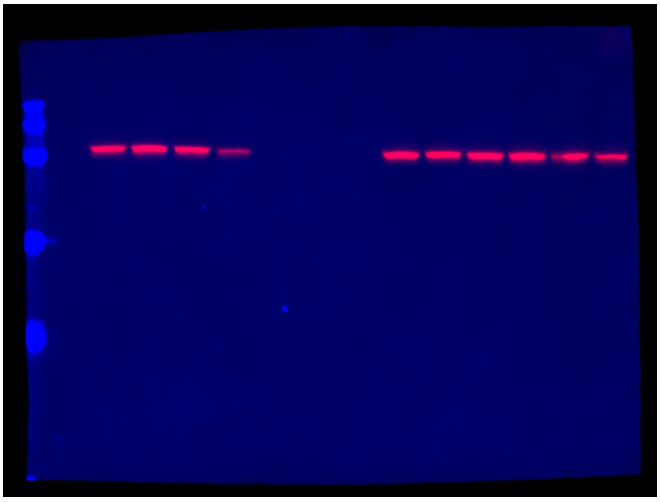

T cells, IL-2, total ERK1/2

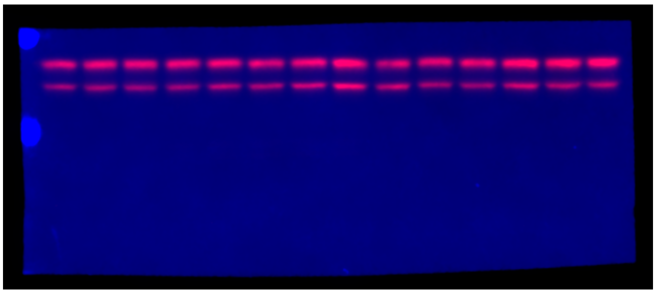

Blots used to generate figure 6

A IL-4, phospho-STAT6

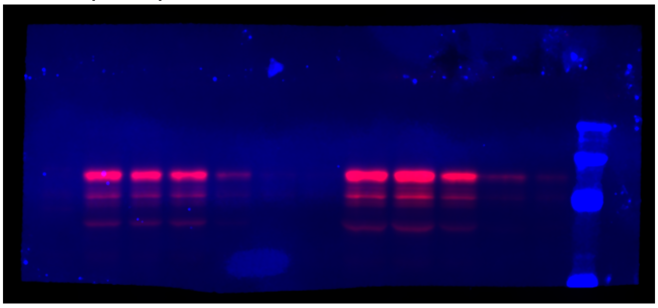

IL-4, total ERK1/2

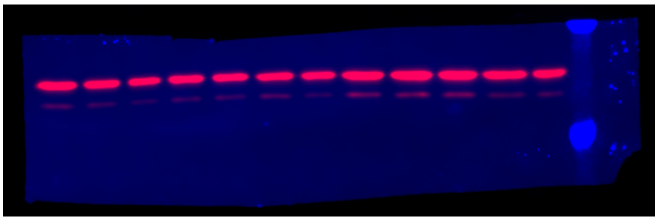

B GM-CSF, phospho-STAT5

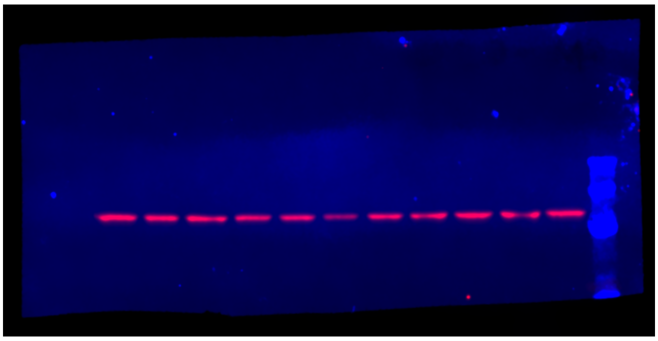

GM-CSF, total ERK1/2

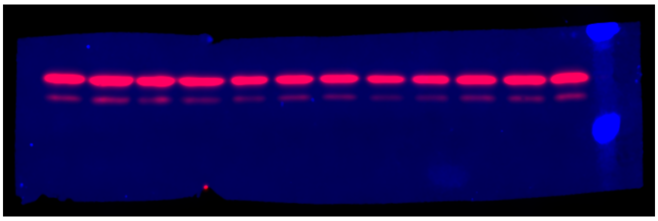

C IL-10, phospho-STAT3

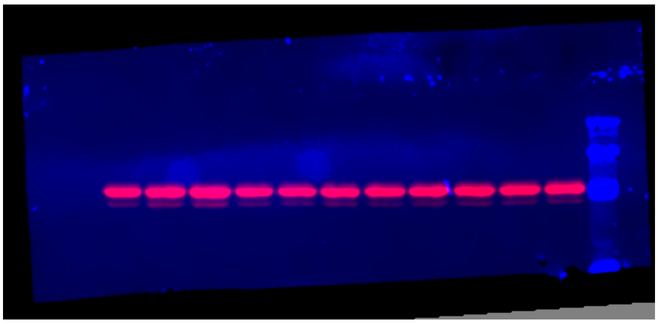

IL-10, total ERK1/2

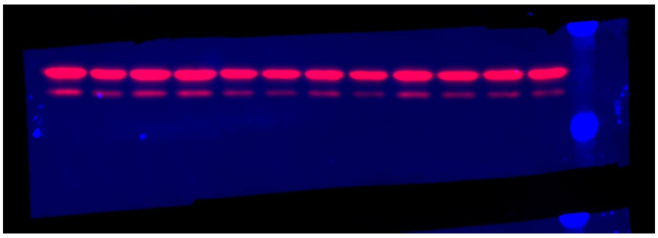

D IFNβ, phospho-STAT1

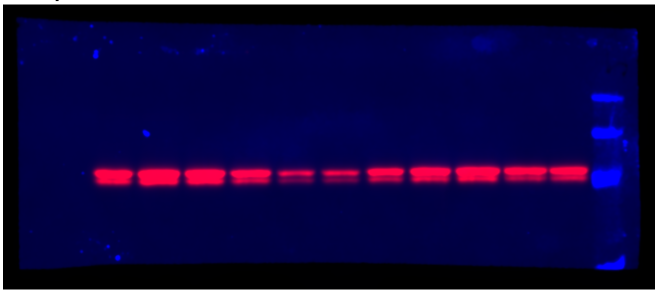

IFNβ, total ERK1/2

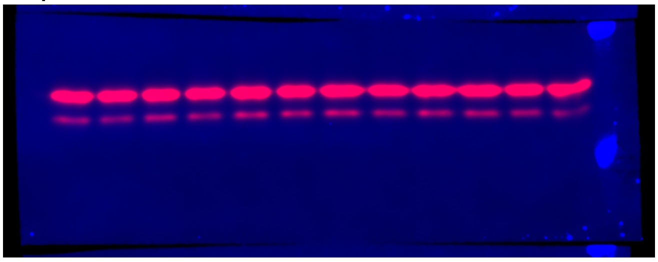

Blots used to generate Supplementary figure XX

phospho-STAT6

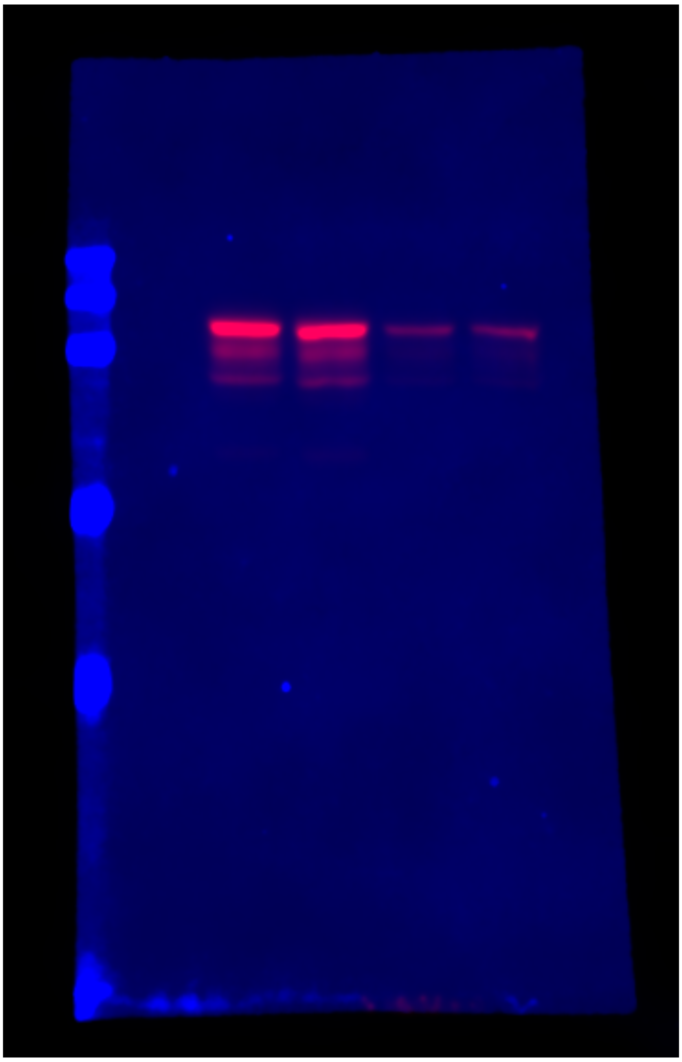

Total ERK1/2

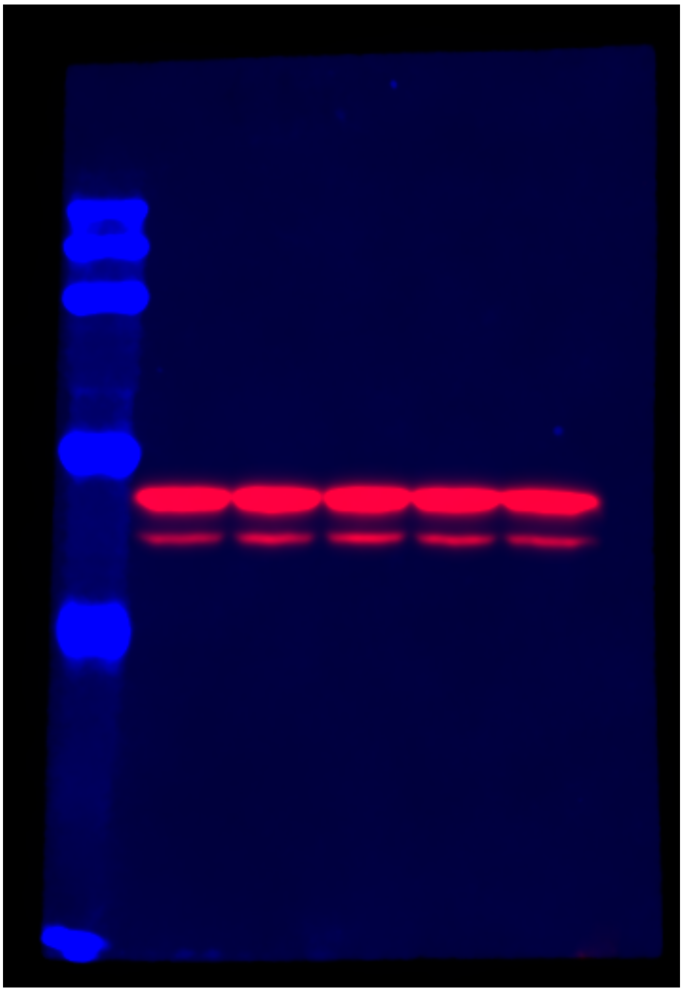

Supplement: Supplementary file 1 — Supplementary Information [file 41598_2021_89356_MOESM1_ESM.pdf]
